# Prediction Models for Obstructive Sleep Apnea in Korean Adults Using Machine Learning Techniques

**DOI:** 10.3390/diagnostics11040612

**Published:** 2021-03-30

**Authors:** Young Jae Kim, Ji Soo Jeon, Seo-Eun Cho, Kwang Gi Kim, Seung-Gul Kang

**Affiliations:** 1 Department of Biomedical Engineering, Gil Medical Center, Gachon University College of Medicine, Incheon 21565, Korea; youngjae@gachon.ac.kr (Y.J.K.); jeon1923@naver.com (J.S.J.); 2 Department of Psychiatry, Gil Medical Center, Gachon University College of Medicine, Incheon 21565, Korea; arztin01@gilhospital.com

**Keywords:** obstructive sleep apnea, machine learning, predict model, logistic regression, support vector machine, random forest, XGBoost

## Abstract

This study aimed to investigate the applicability of machine learning to predict obstructive sleep apnea (OSA) among individuals with suspected OSA in South Korea. A total of 92 clinical variables for OSA were collected from 279 South Koreans (OSA, *n* = 213; no OSA, *n* = 66), from which seven major clinical indices were selected. The data were randomly divided into training data (OSA, *n* = 149; no OSA, *n* = 46) and test data (OSA, *n* = 64; no OSA, *n* = 20). Using the seven clinical indices, the OSA prediction models were trained using four types of machine learning models—logistic regression, support vector machine (SVM), random forest, and XGBoost (XGB)—and each model was validated using the test data. In the validation, the SVM showed the best OSA prediction result with a sensitivity, specificity, and area under curve (AUC) of 80.33%, 86.96%, and 0.87, respectively, while the XGB showed the lowest OSA prediction performance with a sensitivity, specificity, and AUC of 78.69%, 73.91%, and 0.80, respectively. The machine learning algorithms showed high OSA prediction performance using data from South Koreans with suspected OSA. Hence, machine learning will be helpful in clinical applications for OSA prediction in the Korean population.

## 1. Introduction

Obstructive sleep apnea (OSA) is a common sleep disorder that occurs in approximately 14% of adult men and 5% of adult women [1,2]. It is characterized by the repetitive cessation of airflow during sleep due to upper airway obstruction [3]. The symptoms of OSA include frequent snoring, witnessed apnea, choking or snorting during sleep, frequent awakening, non-refreshing sleep, and excessive daytime sleepiness [4]. This disease could be a risk factor for the development of cardiovascular diseases, such as hypertension, atherosclerosis, coronary heart disease, and cerebrovascular disease [5]. In addition, OSA is related to diabetes mellitus, insulin resistance, dyslipidemia, obesity, and cognitive disorders [6,7,8].

Considering the high prevalence of OSA and its impact on health and quality of life, early diagnosis is critical. OSA is diagnosed when the patient has an apnea–hypopnea index (AHI) ≥ 5/h and symptoms of OSA, or an AHI ≥ 15 according to the international diagnostic criteria [9]. Therefore, overnight polysomnography (PSG) is essential for an official diagnosis of OSA. However, since PSG is expensive, physicians are hesitant to recommend PSG to all patients who snore, and the patients often refuse to undergo the OSA diagnostic process.

Therefore, to prioritize patients with suspected OSA, many studies have attempted to develop a prediction model for OSA and AHI that does not require PSG [10,11,12,13,14]. Most of these prediction models used regression analyses based on demographic characteristics, clinical symptoms, and anthropometric variables, such as body mass index and the circumference of the neck or waist [10,11,12,13,14]. Many of the studies have been conducted in Western countries, but previous prediction model studies showed significantly different results according to the ethnicity, country, and clinical characteristics of the participants [10,11,13,14]. For example, in East Asians such as Koreans, Chinese, and Japanese, OSA is relatively common, even in non-obese individuals, due to narrower craniofacial characteristics [15]. Therefore, OSA prediction models need to be performed individually in as many ethnic groups as possible. With the recent development of machine learning techniques, these techniques are increasingly being utilized in prediction model studies.

Machine learning is a field of artificial intelligence and is a technology wherein computers learn from empirical data and identify a series of hidden regularities in them. Conventional data analysis is performed through a one-time procedure based on the opinion of an analyst and systematized in the form of a fixed model. Conversely, machine learning can automatically perform continuous and repetitive learning, leading to a gradual increase in performance [16,17]. Machine learning has strengths in areas that are difficult to define clearly based on mathematical knowledge, such as disease identification in the medical field. Furthermore, the medical field, where a large amount of data can be obtained, is one of the best areas to apply machine learning, as this method requires a large amount of data [18]. Various machine learning algorithms depend on the required learning method and purpose. For prediction models, classification models of supervised learning, which provide labeled data in training, and various models such as logistic regression (LR), support vector machine (SVM), random forest (RF), and XGBoost (XGB) are commonly used [19,20,21,22,23,24,25,26,27,28,29,30,31].

Existing OSA prediction model studies have used regression analysis or SVM [14,24,30,31]. Francisco et al. conducted a study on OSA prediction based on LR using the data of 433 persons and reported a sensitivity of 74.6% and a specificity of 66.3% in correctly predicting a diagnosis of OSA in their participants [14]. Chen et al.’s OSA prediction model was based on SVM using the data of 566 persons and obtained a sensitivity of 83.51% [24]. However, regression analysis and SVM have not been used frequently in recent years. Hence, a performance comparison with the latest models is required. In addition, no OSA prediction model using machine learning has been reported in South Korea.

This study aimed to train and compare OSA prediction models based on four machine learning algorithms (LR, SVM, RF, and XGB) using data from a South Korean population. Thus, this study investigated the applicability of various machine learning algorithms for OSA prediction and determined which was the most optimal.

## 2. Materials and Methods

### 2.1. Ethics Statement

Written informed consent was obtained from all participants, and this study was approved by the Institutional Review Boards of Gil Medical Center (GIRBA2764-2012, approved on 29 May 2012) and Daegu Catholic University Medical Center (CR-11-063, approved on 15 June 2011).

### 2.2. Participants and Data Collection

We collected clinical data from 285 participants from the Gil Medical Center and Daegu Catholic University Medical Center. All participants were Koreans who had OSA symptoms, which include frequent snoring, witnessed apnea during sleep, and daytime sleepiness. The clinical data consisted of 92 clinical variables, including answers to questionnaires that are highly correlated with OSA and sleep status. The questionnaires included the Korean versions of the Berlin questionnaire (BQ) [32], Epworth Sleepiness Scale (ESS) [33], Pittsburgh Sleep Quality Index (PSQI) [34], and Fatigue Severity Scale (FSS) [35]. All anthropometric measurements were performed before PSG. The measurements included weight, height, neck circumference, waist circumference, buttock circumference, and facial surface measurements (distances among the nasion, subnasale, stomion, menton, cervicale, and ideal menton) [36].

All participants underwent attended full-channel nocturnal PSG. The PSG results were scored according to the American Academy of Sleep Medicine (AASM) recommendations [37]. AHI was determined using the recommended hypopnea rules in the AASM manual. The cut-off for OSA was defined as an AHI ≥ 5/h, and those with an AHI < 5/h were defined as not having OSA.

**Figure 1:**
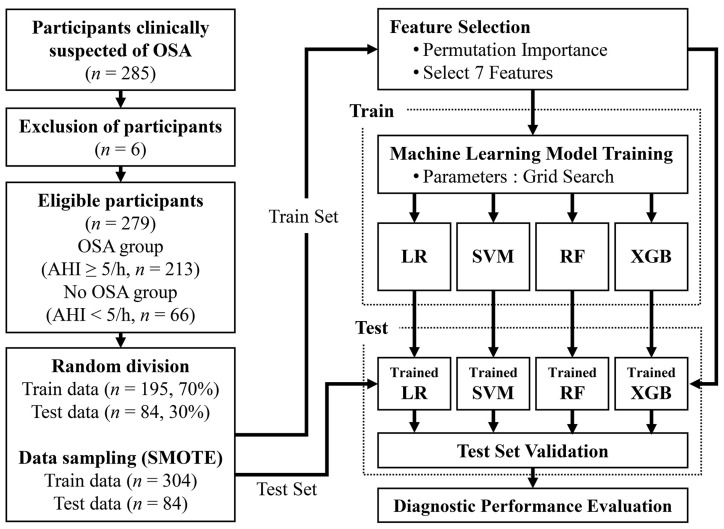
Flowchart of obstructive sleep apnea (OSA) data collection and analysis. Train data: OSA (*n* = 149), no OSA (*n* = 46); Test data: OSA (*n* = 64), no OSA (*n* = 20). OSA, obstructive sleep apnea; AHI, apnea–hypopnea index; CV, cross-validation; SMOTE, synthetic minority oversampling technique.

The clinical data of 279 patients were used in the experiment, except for six patients with missing values in the collected data. The participants were classified into two groups (213 participants with OSA and 66 participants without OSA). More detailed inclusion and exclusion criteria, collected clinical information, and anthropometric measurements, and the implementation of PSG are described in a previous paper published using these participants [36]. Figure 1 shows a flowchart of the OSA data collection and analysis.

### 2.3. Feature Selection

**Figure 2:**
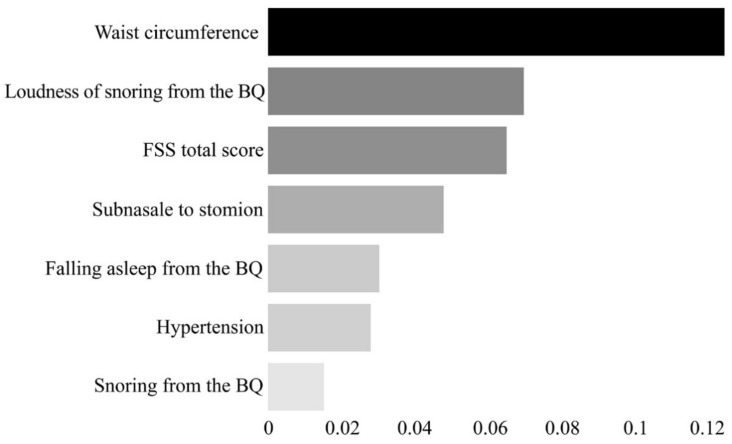
Feature importance plot for the seven final features selected through the permutation feature importance algorithm. The x-axis represents the importance of each feature. The scale of the x-axis is not absolute; it represents the magnitude of the relative importance. BQ, Berlin Questionnaire; FSS, Fatigue Severity Scale.

Here, the permutation feature importance algorithm was used for 92 features (i.e., clinical variables) to calculate the importance of each feature. The importance is calculated by measuring the change of a certain score when the index of each feature is randomly shuffled to the extent that the model has been trained once in the initial stage [38,39]. As a result, seven features were selected: hypertension, waist circumference, length between the subnasale and stomion (subnasale to stomion), snoring from the BQ, loudness of snoring from the BQ, frequency of falling asleep (falling asleep from the BQ), and the FSS total score (Figure 2).

### 2.4. Machine Learning Models to Predict OSA

To train the machine learning model, the dataset was divided into training data and test data. In each of the OSA and non-OSA groups, 30% of the data were randomly selected and used as the test data (OSA, *n* = 64; no OSA, *n* = 20). The remaining data were used as training data (OSA, *n* = 149; no OSA, *n* = 46).

Four machine learning models were trained: LR, SVM, RF, and XGB. LR is a statistical technique used to predict the probability of an event using a linear combination of independent variables, and it is an algorithm that classifies values by applying a logistic function to coefficients calculated by linear regression [19]. SVM is a machine learning technique that converts input data into a high-dimensional space to find an optimal decision boundary that maximizes the margin between data groups [22]. RF is an ensemble model that has an extended form of the decision tree technique. RF is a machine learning technique that forms multiple decision trees and determines the best classification performance results from the results classified by each tree [27]. XGB is an algorithm created by compensating for the disadvantages of the gradient boosting model (GBM) [40]. XGB has a faster execution time than GBM, superior prediction performance compared to other models, and the risk of overfitting is low owing to the overfitting regulation function [28]. The optimal parameters for each machine learning method were selected through a grid search [41] (Table 1).

### 2.5. Statistical Analysis

The predictive performance of LR and the three other machine learning techniques (SVM, RF, and XGB) are presented in terms of the accuracy, sensitivity, specificity, positive predictive value (PPV), and negative predictive value (NPV), calculated based on true positive, true negative, false positive (FP), and false negative (FN). In addition, the area under the receiver operating characteristic curve (AUC) for each machine learning model was calculated to evaluate the general prediction performance.

The machine learning models and diagnostic performance were evaluated using the open-source statistical software Python (version 3.7.0; Python Software Foundation, Wilmington, DE, USA) and scikit-learn library version 0.23.2 [42,43]. Statistical analysis of the receiver operating characteristic (ROC) and ROC comparison analysis was performed using MedCalc (MedCalc Software Ltd., Mariakerke, Belgium) version 14.0. Statistical analysis of the clinical data was performed using SPSS for Windows (version 23; IBM Corp., Armonk, NY, USA). Statistical significance was set at *p* < 0.05.

## 3. Results

The demographic and clinical characteristics of the participants and comparisons between the OSA and non-OSA groups are presented in Table 2.

**Figure 3:**
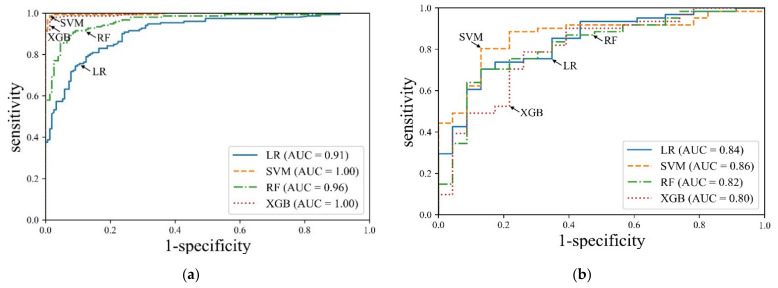
Comparison of receiver operation characteristics between different machine learning models for OSA prediction. The SVM model shows the highest AUC. (**a**) ROC curve obtained with the training data, and (**b**) ROC curve obtained with the test data. LR, logistic regression; SVM, support vector machine; RF, random forest; XGB, XGBoost; OSA, obstructive sleep apnea; AUC, area under the curve; ROC, receiver operating characteristic.

In this study, we trained each machine learning model based on the seven selected features and compared the OSA prediction performance based on the test data created separately. Table 3 and Figure 3 show the OSA prediction performance of each machine learning model.

For the machine learning models LR, SVM, RF, and XGB were trained using the training data of OSA prediction, the accuracy was 83.2% (confidence interval [CI]: 78.5–87.3), 98.0% (CI: 95.8–99.3), 90.8% (CI: 87.0–93.8), and 97.0% (CI: 94.5–98.6), respectively, and the AUC was 0.91 (CI: 0.87–0.94), 0.99 (CI: 0.98–1.0), 0.96 (CI: 0.94–0.98), and 0.99 (CI: 0.97–1.0), respectively. That is, every machine learning model was trained with excellent performance.

The results of using the separately created test data to validate the OSA prediction performance of each machine learning model that completed training indicated that the SVM model showed the best performance, with 80.33% sensitivity (CI: 64.36–83.81), 86.96% specificity (CI: 66.4–97.2), 88.52% PPV (CI: 80.51–93.51), 69.57% NPV (CI: 51.98–82.84), and 83.33% accuracy (CI: 73.62–90.58). On the other hand, the XGB model showed the lowest performance with a 78.69% sensitivity (CI: 66.3–88.1), 73.91% specificity (CI: 51.6–89.8), 85.71% PPV (CI: 77.16–91.42), 53.57% NPV (CI: 39.56–67.04), and 75.0% accuracy (CI: 64.36–83.81). The performance ranking of the machine learning algorithms in terms of accuracy was as follows: SVM, RF, LR, and XGB.

In the ROC analysis, the highest AUC was obtained by the SVM (0.87, CI: 0.77–0.93), followed by the LR (0.84, CI: 0.74–0.91), RF (0.82, CI: 0.72–0.89), and XGB (0.80, CI: 0.70–0.88) (Figure 3). There was no significant difference between AUCs (*p* = 0.37).

**Figure 4:**
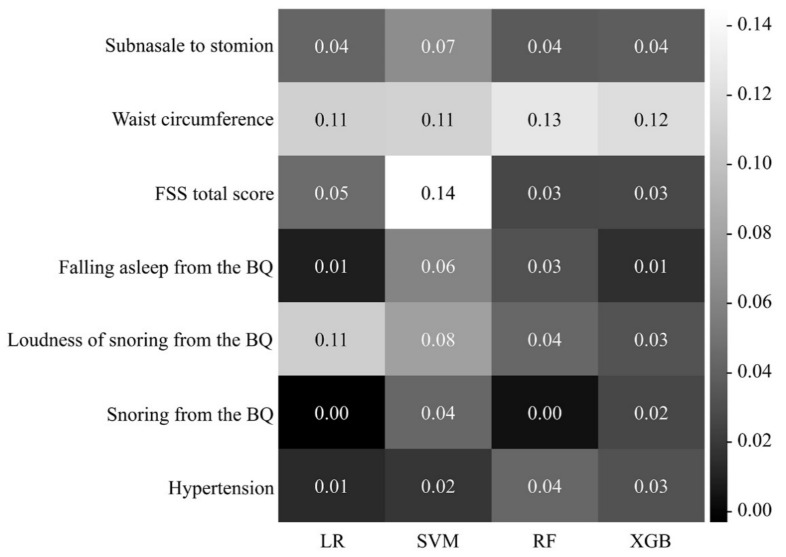
Heatmap for the effect of selected features on OSA prediction in each machine learning algorithm. The higher the value (the closer the color is to white), the larger the effect of the feature on OSA prediction. FSS, Fatigue Severity Scale; BQ, Berlin Questionnaire; OSA, obstructive sleep apnea.

Figure 4 shows a heatmap of the effect of the seven features on OSA prediction in each model. In every model, the variables that had the greatest effect on OSA prediction were waist circumference (LR, 0.11; RF, 0.13; XGB, 0.12; and SVM, 0.11) and Berlin loudness of snoring (LR, 0.11; RF, 0.04; XGB, 0.03; and SVM, 0.07).

**Figure 5:**
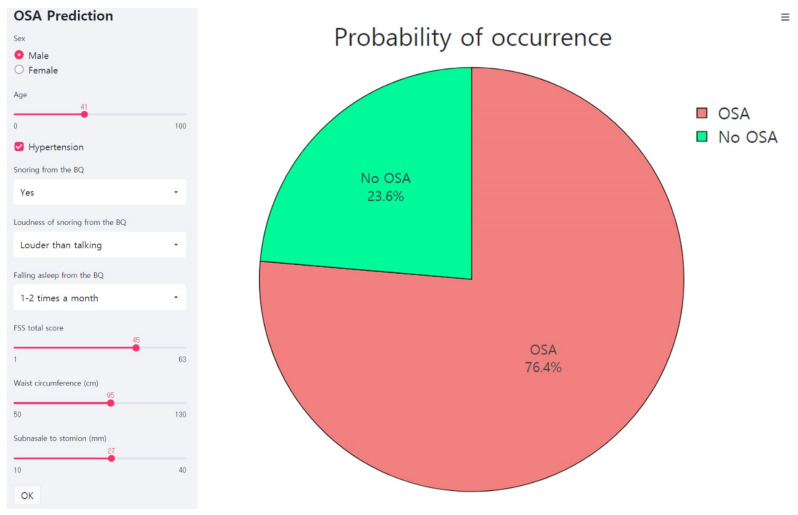
Web page-based application for the OSA prediction. This is an example of inputting data of a male participant into an application. The application predicts the probability of the OSA as 76.4%. FSS, Fatigue Severity Scale; BQ, Berlin Questionnaire; OSA, obstructive sleep apnea.

Figure 5 shows the web page-based application for the OSA prediction. The machine learning model as developed here is linked to the web page and the probability for the OSA is provided when seven features are entered into the application.

## 4. Discussion

In this study, we selected significant clinical indices for predicting OSA based on 92 clinical variables from 279 individuals and then compared the OSA prediction performance of different machine learning algorithms (i.e., LR, SVM, RF, and XGB).

Seven clinical indices (i.e., hypertension, waist circumference, subnasale to stomion, snoring from the BQ, loudness of snoring from the BQ, falling asleep from the BQ, and FSS total score) were found to have a significant effect on OSA prediction. The OSA prediction performance of the LR, SVM, RF, and XGB models trained using the selected indices as inputs were ranked based on the AUC as follows: SVM (0.87), LR (0.84), RF (0.82), and XGB (0.80). However, based on accuracy, the ranking was SVM (83.33%), RF (78.57%), LR (75.0%), and XGB (75.0%). Most of the models used in this experiment tended to have higher specificity than sensitivity, and while the PPV was high, the NPV was low. This result implies that the majority of models had few FPs and many FNs. FP refers to a case in which the prediction model mispredicts the existence of OSA (i.e., OSA group) even when the actual case is that OSA does not exist (i.e., no OSA group), whereas FN refers to a case in which the prediction model mispredicts the absence of OSA (i.e., no OSA group) even when the actual case is that OSA does exist (i.e., OSA group). In general, when the weight of the training data is leaning toward one side between the two groups, the training of the prediction model is often biased toward the group with more training data. In this study, there was a risk of training biased toward the OSA group because this group had three times as much training data as the no OSA group. However, the prediction results in most models did not show a large deviation between sensitivity and specificity (*p* = 0.08) and were not biased toward the non-OSA group. Therefore, it was interpreted that the data used in the training represented the OSA group and were suitable for machine learning.

In the current experiment, the older SVM model performed better than the latest XGB and RF models. This is because SVM exhibits good performance with small datasets. The drawback of SVM is that the accuracy drops when there are many overlaps of boundaries between data clusters. Therefore, when the boundary between the data for prediction is ambiguous, the accuracy decreases as the number of data points increases. In the case of OSA prediction, good performance was observed because there were clear differences in features between the OSA and non-OSA groups, and the amount of data was not large. However, in the future, this should be investigated by collecting more data to ensure that clear differences in features exist between the OSA and the no OSA groups because the number of training data in this study was somewhat small.

Among the seven selected features, waist circumference and loudness of snoring from the BQ had a strong effect on OSA prediction. Snoring is a core sign and one of the most important clinical symptoms of OSA. Loudness of snoring usually correlates with the severity of OSA (i.e., AHI) [44]. Waist circumference is also an important risk factor and predictor of OSA, along with body weight, body mass index, and neck circumference, and is significantly correlated with OSA severity [45]. In a previous study, this correlation was reported in Koreans [12]. In past OSA prediction model studies, waist circumference [11,12,30,31] and loudness of snoring [11,30] were suggested as important features for the prediction of OSA.

Here, we created OSA prediction models using data from individuals with suspected OSA from South Korea based on four types of machine learning algorithms, including the most recent algorithms, and we compared their prediction performances to investigate the applicability of machine learning to predict OSA. The four types of machine learning models showed high accuracies of over 80%, thereby confirming sufficient potential for utilizing machine learning in OSA prediction. In addition, it was found that SVM was the best model for OSA prediction for small datasets. However, a limitation of this study is the small dataset used in the experiment. The total dataset was insufficient to train and validate the machine learning models, and because the data ratio between the OSA and non-OSA groups was biased, there remains a reasonable doubt about the performance of the models.

In addition, validation of the overfitting was insufficient in the process of training and validating the model. The test data were randomly selected and used in the validation process, but there remains a risk of bias in that the validation result may be different when other randomly selected data are used as test data. Certainly, the possibility of overfitting is low as the total dataset is small, and a similar performance was observed in both the training data and the test data. However, an additional analysis of overfitting is required for accurate validation. Therefore, in the future, more data should be collected to further train the machine learning models, and additional analysis of overfitting should be conducted through validation methods, such as cross-validation and external validation. In the future, if the data size is increased and further analysis is conducted, the performance of XGB and RF, in addition to SVM, is likely to be improved. However, it is certain that the LR, SVM, RF, and XGB machine learning algorithms showed sufficient potential for OSA prediction using data from South Korea. This suggests that machine learning can play an important role in OSA prediction in clinical settings.

This study is significant in terms of the research process and results compared to previous studies. Unlike previous cases that only attempted OSA prediction in one model such as LR or SVM [14,24], we applied and compared various machine learning methods and proposed the most appropriate machine learning method for OSA prediction. Moreover, in terms of performance, the LR-based OSA prediction model proposed by the Spanish group showed an AUC of 0.78 [14], while the OSA prediction model proposed in this study showed an AUC of 0.87. The OSA prediction model proposed by the Taiwanese group used the same machine learning model as the SVM proposed in this study and showed an accuracy of 87.72% [24], which was higher than the accuracy of 83.33% from the model we proposed. However, the OSA prediction model proposed by the Taiwanese group exhibited a very large deviation, with sensitivity and specificity of 42.86% and 94%, respectively, and it had a very low sensitivity [24]. This implies that learning or training was biased to the non-OSA group, thereby indicating that learning did not take place appropriately. This may be due to the ratio of data composition between OSA and non-OSA groups, methods used in feature selection, or the unoptimized parameters of the learning models. In contrast, our proposed OSA prediction model can be considered a better OSA prediction model because the sensitivity and specificity in the same SVM model were 80.33% and 86.96%, respectively, with little deviation and stable prediction performance. However, we used a smaller number of data when compared to other studies, which can be considered as a limitation of this study. Therefore, further studies with more extensive data collection are required.

Machine learning techniques have the potential to be of key help in the development of digital healthcare, such as mobile applications for the personalized monitoring of OSA in the future. If physiological data such as oxygen saturation, snoring sound, breathing pattern, and heart rate during sleep recorded by wearable devices or mobile phones and clinical information such as hypertension and anthropometric data are combined and analyzed using machine learning methods, the daily monitoring of OSA risk and progress of AHI will be possible. To make machine learning methods more robust, replication studies for machine learning methods in participants with suspected OSA using big data are needed in the future.

## 5. Conclusions

In this study, the OSA prediction models were trained using four types of machine learning models: logistic regression, SVM, random forest, and XGB, and each model was validated. All four models showed high OSA prediction performance using data from South Koreans with suspected OSA, and SVM showed the best OSA prediction result. In the future, machine learning techniques are expected to be critical in developing clinically useful digital healthcare for OSA and other sleep disorders.

## Figures and Tables

**Table 1:** Parameters for each machine learning model are selected through the grid search.

Model	Parameters
LR	C: 1, penalty: l2, random_state: 80
SVM	C: 10, gamma: 0.1, probability: True, random_state: 80
RF	max_depth: 6, min_samples_split: 25, *n*_estimators: 400, random_state: 80
XGB	learning_rate: 0.01, max_depth: 4, *n*_estimators: 400, random_state: 80

LR, logistic regression; SVM, support vector machine; RF, random forest; XGB, XGBoost. C: inverse of regularization strength; penalty: used to specify the norm used in the penalization; random_state: use a new random number generator seeded by the given integer; gamma: kernel coefficient for ‘radial basis function,’ ‘poly,’ and ‘sigmoid’; probability: whether to enable probability estimates; max_depth: the maximum depth of the tree; min_samples_split: the minimum number of samples required to split an internal node; *n*_estimators: the number of trees in the forest; learning_rate: shrinks the contribution of each tree by learning_rate.

**Table 2:** Characteristics of the participants in the OSA and no OSA groups and the comparison between the groups.^1^

Variables	OSA (*n* = 213)	No OSA (*n* = 66)	Statistics
**Age**	45.4 ± 10.9	41.8 ± 11.9	*p* = 0.0254
**Sex**			*p* = 0.063
Male	184 (86.4%)	50 (75.8%)	
Female	29 (13.6%)	16 (24.2%)	
**Body mass index (** **kg/m^2^)**			*p* = 0.0005
0–18.4	2 (0.9%)	0 (0.0%)	
18.5–22.9	28 (13.1%)	23 (34.8%)	
23–24.9	49 (23.0%)	18 (27.3%)	
25–29.9	110 (51.6%)	22 (33.3%)	
≥ 30	24 (11.3%)	3 (4.5%)	
**Hypertension**	57 (26.8%)	7 (10.6%)	*p* = 0.0105
**ESS total score**	10.0 ± 4.7	8.3 ± 4.4	*p* = 0.016
**FSS total score**	35.1 ± 12.1	38.3 ± 12.3	*p* = 0.064
**Berlin questionnaire**			
**Loudness of snoring**			*p* < 0.00001
Louder than breathing	7 (3.3%)	11 (16.7%)	
Loud as talking	28 (13.1%)	17 (25.8%)	
Louder than talking	54 (25.4%)	17 (25.8%)	
Can be heard in adjacent rooms	124 (58.2%)	21 (31.8%)	
**Frequency of snoring**			*p* = 0.0299
Never or nearly never	1 (0.5%)	3 (4.5%)	
1–2 times a month	2 (0.9%)	1 (1.5%)	
1–2 times a week	9 (4.2%)	4 (6.1%)	
3–4 times a week	16 (7.5%)	10 (15.2%)	
Nearly every day	185 (86.9%)	48 (72.7%)	
**Quit breathing**			*p* = 0.0103
Never or nearly never	18 (8.5%)	8 (12.1%)	
1–2 times a month	11 (5.2%)	6 (9.1%)	
1–2 times a week	22 (10.3%)	9 (13.6%)	
3–4 times a week	47 (22.1%)	24 (36.4%)	
Nearly every day	115 (54.0%)	19 (28.8%)	
**Tiredness**			*p* = 0.0675
Never or nearly never	5 (2.3%)	4 (6.1%)	
1–2 times a month	10 (4.7%)	5 (7.6%)	
1–2 times a week	40 (18.8%)	5 (7.6%)	
3–4 times a week	71 (33.3%)	18 (27.3%)	
Nearly every day	87 (40.8%)	34 (51.5%)	
**Falling asleep**			*p* = 0.0114
Never or nearly never	30 (14.1%)	9 (16.6%)	
1–2 times a month	51 (23.9%)	20 (30.3%)	
1–2 times a week	82 (38.5%)	34 (51.5%)	
3–4 times a week	29 (13.6%)	3 (4.5%)	
Nearly every day	21 (9.9%)	0 (0.0%)	
**Anthropometric measurements**			
**Neck circumference (cm)**			*p* < 0.0001
< 30	0 (0%)	6 (9.1%)	
30.1–35	36 (16.9%)	12 (18.2%)	
35.1–40	118 (55.4%)	45 (68.2%)	
40.1–45	56 (26.3%)	3 (4.5%)	
≥ 45.1	3 (1.4%)	0 (0%)	
**Waist circumference (cm)**			*p* < 0.0001
< 70	1 (0.5%)	2 (3.0%)	
70.1–80	17 (8.0%)	18 (27.3%)	
80.1–90	86 (40.4%)	34 (51.5%)	
90.1–100	84 (39.4%)	9 (13.6%)	
100.1–110	19 (8.9%)	2 (3.0%)	
110.1–120	6 (2.8%)	1 (1.5%)	
**Subnasale to stomion (mm)**			*p* = 0.0428
< 20	0 (0%)	1 (1.5%)	
20.1–25	55 (25.8%)	27 (40.9%)	
25.1–30	129 (60.6%)	33 (50.0%)	
30.1–35	27 (12.7%)	5 (7.6%)	
≥ 40	2 (0.9%)	0 (0%)	
**Polysomnography data**			
**AHI (number of events per hour)**	32.3 ± 22.5	1.9 ± 1.3	*p* < 0.001
**O2 desaturation index (number of events per hour)**	27.1 ± 20.5	1.7 ± 1.4	*p* < 0.001
**Total sleep time (minutes)**	340.6 ± 54.9	343.9 ± 52.0	*p* = 0.660

**Table 3:** The observed AUC, accuracy, sensitivity, specificity, PPV, and NPV for OSA prediction of each machine learning model for the training and test data.^1^

	AUC (95% CI)	Accuracy (%) (95% CI)	Sensitivity (%) (95% CI)	Specificity (%) (95% CI)	PPV (%) (95% CI)	NPV (%) (95% CI)
**Training dataset**						
LR	0.91 (0.87–0.94)	83.22 (78.54–87.25)	80.92 (73.76–86.83)	85.53 (78.91–90.70)	84.83 (79.04–89.24)	81.76 (76.25–86.23)
SVM	0.99 (0.98–1.00)	98.03 (95.75–99.27)	96.71 (92.49–98.92)	99.34 (96.39–99.98)	99.32 (95.42–99.90)	96.79 (92.73–98.62)
RF	0.96 (0.94–0.98)	90.79 (86.96–93.79)	90.79 (85.03–94.87)	90.79 (85.03–94.87)	90.79 (85.65–94.21)	90.79 (85.65–94.21)
XGB	0.99 (0.98–1.00)	97.04 (94.45–98.64)	96.71 (92.49–98.92)	97.37 (93.40–99.28)	97.35 (93.32–98.98)	96.73 (92.59–98.59)
**Test dataset**						
LR	0.84 (0.74–0.91)	75.00 (64.36–83.81)	70.49 (57.4–81.5)	86.96 (66.4–97.2)	88.46 (76.56–95.65)	53.13 (34.74–70.91)
SVM	0.87 (0.77–0.93)	83.33 (73.62–90.58)	80.33 (68.2–89.4)	86.96 (66.4–97.2)	88.52 (80.51–93.51)	69.57 (51.98–82.84)
RF	0.82 (0.72–0.89)	78.57 (68.26–86.78)	70.49 (57.4–81.5)	86.96 (66.4–97.2)	86.44 (78.28–91.86)	60.00 (44.15–74.00)
XGB	0.80 (0.70–0.88)	75.00 (64.36–83.81)	78.69 (66.3–88.1)	73.91 (51.6–89.8)	85.71 (77.16–91.42)	53.57 (39.56–67.04)

LR, logistic regression; SVM, support vector machine; RF, random forest; XGB, XGBoost; OSA, obstructive sleep apnea; AUC, area under the curve; PPV, positive predictive value; NPV, negative predictive value; CI, confidence interval. ^1^ Data are reported as mean ± standard deviation or n (%). AHI, apnea-hypopnea index; ESS, Epworth Sleepiness Scale; Falling asleep, frequency of falling asleep during driving; PSQI, Pittsburgh Sleep Quality Index; Quit breathing, frequency of quitting breathing; Tiredness, frequency of tiredness during waking time; FSS, Fatigue Severity Scale.

## Data Availability

The datasets generated or analyzed during the current study are available from the corresponding author upon reasonable request.
